# Forecasting Time-Series Energy Data in Buildings Using an Additive Artificial Intelligence Model for Improving Energy Efficiency

**DOI:** 10.1155/2021/6028573

**Published:** 2021-07-27

**Authors:** Ngoc-Son Truong, Ngoc-Tri Ngo, Anh-Duc Pham

**Affiliations:** Faculty of Project Management, The University of Danang-University of Science and Technology, 54 Nguyen Luong Bang, Danang, Vietnam

## Abstract

Building energy efficiency is important because buildings consume a significant energy amount. The study proposed additive artificial neural networks (AANNs) for predicting energy use in residential buildings. A dataset in hourly resolution was used to evaluate the AANNs model, which was collected from a residential building with a solar photovoltaic system. The proposed AANNs model achieved good predictive accuracy with 14.04% in mean absolute percentage error (MAPE) and 111.98 Watt-hour in the mean absolute error (MAE). Compared to the support vector regression (SVR), the AANNs model can significantly improve the accuracy which was 103.75% in MAPE. Compared to the ANNs model, accuracy improvement percentage by the AANNs model was 4.6% in MAPE. The AANNs model was the most effective forecasting model among the investigated models in predicting energy consumption, which provides building managers with a useful tool to improve energy efficiency in buildings.

## 1. Introduction

National development, urbanization, and population growth require a growing energy demand. Buildings account for remarkable energy consumption during their operational stages and are responsible for carbon emissions and global warming. Energy performance in buildings is of prime importance all over the world. Buildings should be designed for occupant's comfort while consuming less energy. Thus, energy efficiency is one of the most concerning topics among academic researchers and decision-makers in the energy sector. It plays a remarkable role in targeting a low-carbon economy [1]. National governments have also recognized the benefits of efficient uses of energy in the building sector. The efficient use of energy in buildings strongly affects the building's capability to meet the building green certificates in the green building rating system to reduce carbon emission and greenhouse effects. Thus, energy usage prediction in buildings is necessary for energy planning, management, and conservation.

Various studies have been conducted to improve building energy performance [2–5]. An accurate forecast of the building energy use is a vital issue in smart building applications. Building energy prediction is typically performed by using engineering-based methods and artificial intelligence (AI) approaches. Because the engineering approach applies thermodynamic equations to predict energy consumption in buildings, they are time-consuming and require a high level of expertise to customize and set thermal parameters for energy performance analysis. To perform energy prediction, the engineering method requires detailed information on the building envelope, thermal properties of construction layers and windows, and the heating, ventilation, air-conditioning system.

The AI-based method infers future energy consumption profiles in buildings using historical data [6]. The advantage of the AI-based approach lies in its learning capability to model the relationship between historical data and future data. The AI-based prediction model requires historical data instead of much detailed building information like the engineering methods. It does not require users to poses a deep knowledge of the thermodynamic behavior of buildings. Some studies have proposed AI models for solving the prediction of building energy performance. For example, Song et al. (2020) presented the evolutionary model construction for predicting electricity data in smart buildings [7]. Wang and Srinivasan (2017) presented an in-deep review of AI-based prediction models for predicting energy use in buildings with special attention on ensemble [6]. Huang et al. (2021) applied a deep learning method in developing an energy management system [8]. Jahani et al. (2020) developed a prediction model by integrating a genetic algorithm and numerical moment matching method to predict energy consumption in residential buildings [9].

Analyzing energy structure and electricity use behavior is important to propose an energy-efficient policy in nations [10]. Forecasting results of energy consumption in buildings is the basis for optimizing building performance and reducing energy costs [11]. AI and machine learning (ML) models have been used in the building energy domain [11–15]. A model can predict future data or generate new insights based on learning from historical data. ML models have been used to forecast thermal demands using skin temperatures [12]. Chou and Ngo (2016) analyzed time-series energy data by integrating an ML and an optimization algorithm to identify building energy data patterns [4].

Among various ML models, artificial neural networks (ANNs), support vector regression (SVR), and multiple linear regression are popular [6]. Ganguly et al. (2020) applied the ANNs model to forecast building energy use in a historical art gallery [16]. Saleh et al. (2019) evaluated the performance of ML models in predicting heating and cooling loads in buildings [17]. Their investigated ML models included ANNs, support vector regression (SVR), Gaussian process (GP), random forest, and gradient boosted regression trees (GBRT). Their experimental result revealed that the GBRT obtained the best performance in terms of the root-mean-square error (RMSE) value. They also concluded that the ANNs model was the best fit for complex datasets. The computing time of the ANNs model is faster than other investigated ML modes in their study [17]. Najafzadeh and Oliveto (2020) applied the support vector machine (SVM), multivariate adaptive regression splines (MARS), and random forest (RF) to predict the approach densimetric Froude number at the incipient motion of riprap stones that can protect rivers from erosion problems [18].

The various ML models were used in [19] to infer data of CO2, TVOC, and HCHO in buildings, which include the SVM, GP, M5P, and backpropagation neural network. The ANNs have been widely used in the energy domain. Sharifa and Hammad (2019) applied ANNs models to select energy renovation methods in buildings concerning energy usage, life cycle cost, and life cycle assessment [20]. The ANNs model was applied to forecast short-term load in buildings [21].

Though these methods can yield a significant proven forecasting accuracy improvement in some cases, they have usually focused on the improvement of the accuracy without paying special attention to the interpretability. Recently, expert systems, mainly developed by means of linguistic fuzzy rule-based systems, allow us to deal with the system modeling with good interpretability [14]. However, these models have strong dependency on an expert and often cannot generate good accuracy. Therefore, combination models, based on the popular methods, expert systems, and other techniques, are proposed to satisfy both high accurate level and interpretability.

Although ML models have been applied in the building energy domain and yielded good forecasting accuracy, the improvement of their performance in energy prediction is still necessary. In addition, the energy consumption in the residential buildings equipped with the solar photovoltaic system has not been investigated in the literature. Therefore, this study proposed the additive artificial neural networks (AANNs) that can accurately predict energy consumption in residential buildings with the renewable energy system. The contributions of this study include (1) collection of building energy consumption profiles in hourly resolution and their associated weather data, (2) investigation of the potential power of artificial intelligence techniques in predicting future building energy consumption, and (3) development of the effectiveness and capability of the AANNs in the prediction of building energy consumption.

The rest of the study is organized as follows.  Section 2 contains the literature review.  Section 3 presents the methodology.  Section 4 contains the results and discussion.  Section 5 shows the conclusions.

## 2. Literature Review

Buildings are responsible for about 30% of the total energy consumption. Energy consumption prediction in buildings is imperative in energy management and conservation because it facilitates a process to assess energy efficiency, perform commissioning, and detect and diagnose building system anomalies [6]. Energy performance in buildings is affected by various uncertainty factors such as heating ventilation air-conditioning (HVAC) systems, building envelops characteristics, and building operating schedules. Uncertainty analysis has been used widely in assessing building energy performance [22–24] since the inherent uncertainty of occupant behavior, the building thermal property, HVAC system, and weather conditions. Four perspectives for assessing building performance have been presented in [22] including uncertainty data sources, forward and inverse methods, application of uncertainty analysis, and available software.

**Figure 1:**
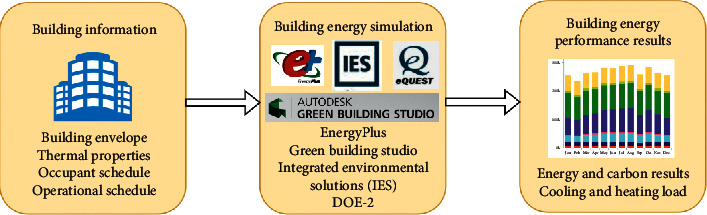
Forward uncertainty analysis for building energy assessment.

Forward and inverse uncertainty analyses are common ways in energy assessment in buildings [22]. The former analysis mainly purposes on quantifying the variation of outputs propagated from the uncertainty from inputs via mathematical models as visualized in Figure 1. This approach can be used to predict energy consumption or energy-efficient design using building energy models (e.g., EnergyPlus or DOE). In contrast, the later analysis aims to determine unknown variables through mathematical models from the measurement and verification process. This approach is also called model calibration that is often used for retrofitting and maintaining buildings. Literature reveals that the forward uncertainty analysis has been used in assessing the building energy performance more than the inverse uncertainty analysis [22].

Building energy efficiency is extremely important in the sustainability of energy and the environment. Chou et al. (2016) presented a smart decision system based on big data analytics and cloud computing for energy efficiency in buildings [4]. Hartono et al. (2020) analyzed modern energy projects in Indonesia and confirmed that modern energy access is one of the main factors of energy spending, especially for low-income households and rural areas [25]. Shaikh et al. (2017) presented a comprehensive review of building energy scenarios, the policy perspectives, and building energy efficiency programs along with landmark buildings and their characteristics in Malaysia [26]. Their study found that there were inadequate incentives for motivating demand-side management, awareness, ineffective management of quality services, and inadequate legal and regulatory frameworks. They suggested that energy-efficient building designs should be considered to reduce energy use over the building life cycle.

Tian et al. (2019) applied the Bayesian network model to identify the most energy-efficient primary cooling systems [27]. The Bayesian network model was trained using high energy-efficient data in buildings. The trained model was then used to decide the primary cooling systems in buildings. Their findings confirmed the applicability of data-driven building design. Zeng et al. (2019) applied the GP regression [11] for predicting electricity consumption in buildings. Their conclusions were those complex buildings such as hotels and shopping malls, and the GP regression was not better than those of simple models because of the inherent complex energy use patterns.

Chen et al. (2016) developed the electric load prediction model by integrating the fuzzy time series and global harmony search algorithm and SVM that can produce reliable prediction results [28]. Wei et al. (2018) reviewed the data-driven approaches for assessing building energy [22]. Their review confirmed that the data-driven approaches have been used widely in the energy domain such as load predictions, energy pattern profiles, and retrofit solutions. Their investigation revealed that the ANNs model was the most popular in applications from energy prediction to retrofit solutions. The SVM models were often used for large-scale building energy analysis due to their simplicity in the training process.

Amasyali and El-Gohary (2018) reviewed data-driven approaches for predicting building energy consumption [29]. Their study focused on investigating the prediction scopes, the data preprocessing methods, the ML prediction model, and the performance measures used for evaluation. In terms of the prediction scopes, there were two types of buildings which are residential and nonresidential building; five data resolutions are subhourly, hourly, daily, monthly, and yearly. Regarding data size, most of the reviewed studies used a one-month to a one-year dataset. The review indicated that ANNs (47%) and SVM (25%) were the two most popular ML models used for building energy prediction. There is no study that applied ML models for dealing with energy consumption in a residential building that uses a solar photovoltaic system. The solar photovoltaic system can produce energy sources, while buildings are in operation during the daytime. Thus, this study applied the additive approach to improving the performance of ANNs in predicting energy use in building with a solar photovoltaic system.

## 3. Methodology

**Figure 2:**
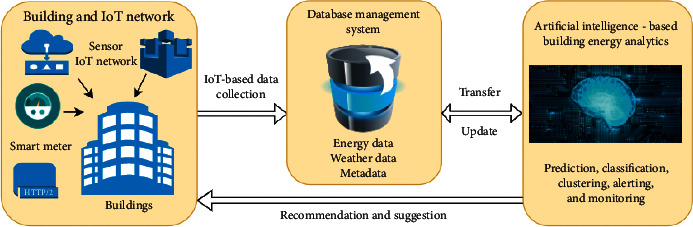
AI techniques for building energy performance assessment.

Figure 2 shows the overall structure of AI applications for assessing energy performance in buildings. This flowchart consists of three components, including the buildings and IoT network, database management system, and AI-based building energy analytics. AI-based building energy analytics may do some tasks such as predictions, classification, clustering, alerting, and monitoring. Results from these tasks can recommend or suggest building users for doing further actions for saving energy and reducing energy costs. This study focuses on the prediction task in which some AI techniques were applied. A mathematical theory of AANNs for energy prediction models in this study is presented in the following.

### 3.1. Artificial Neural Networks

ANNs models have proven their effectiveness for engineering problems [30]. The multilayer perceptron is a feedforward neural network that reflects inputs onto a set of appropriate outputs. The schema of ANN models includes a layer for inputs with sensory input nodes, hidden layers of computation nodes, and an output layer with a computation node. Equation (1) expresses an activated neuron in a hidden layer.

(1) $$\begin{array}{c} {\text{net}}_{j}=\sum w_{ji}x_{i}\text{ and }y_{j}=f\left({\text{net}}_{j}\right), \end{array}$$

where net_*j*_ represents an activation of *j*^th^ neuron, *i* represents a neuron in the preceding layer, w_*ji*_ represents a weight of the relationship between neuron *j* and neuron *i*, *x*_*i*_ represents the output of neuron *i*, and *y*_*j*_ represents the transferring function as

(2) $$\begin{array}{c} f\left({\text{net}}_{j}\right)=\frac{1}{1+e^{\lambda {\text{net}}_{j}}}, \end{array}$$

where *λ* adjusts the function gradient.

Weights *w*_*ji*_were updated during the training process of ANN models as equation (3). Δ_*ji*_(*h*) is the difference between two iterations as equation (4).

(3) wjih=wh−1ji+Δjih,

(4) $$\begin{array}{c} \Delta_{ji}\left(h\right)=\eta \delta_{pi}\chi_{pi}+\alpha \Delta w_{ji}\left(h-1\right), \end{array}$$

where *η* represents a learning rate parameter; *δ*_*pi*_ represents a propagated error; *χ*_*pi*_ represents a output of neuron *i* for record *p*; *α* represents a momentum parameter; and Δw_*ji*_(*h*-1) is a change in *w*_*ji*_ in the previous cycle.

### 3.2. Additive Artificial Neural Networks

The AANNs model is a meta-model that can improve the effectiveness of a classical ANNs model [31]. Gradient boosting was used to construct the additive regression model by sequentially fitting the base learner such as an ANNs model to the current pseudoresiduals by least-squares at each generation. The pseudoresiduals were gradients of the loss functional being minimized. Each generation fits a model to the residuals left by the ANNs on the previous generation. Predictive results are built by adding the predictions of each model. Reducing the shrinkage parameter helps prevent overfitting and has a smoothing effect. Details of the AANN models are present in [31].

**Figure 3:**
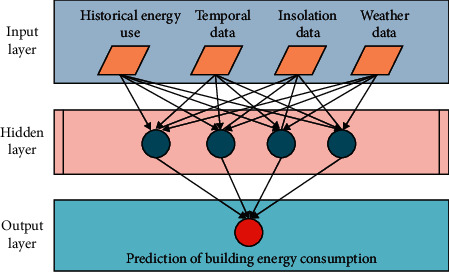
Schema of artificial neural networks.

Figure 3 shows the schema of an ANNs model for building energy use prediction in this study. The input layer contains the historical energy consumption data, temporal data, insolation data, and weather data (e.g., outdoor temperature). The hidden layer was used to perform the transforming computation between inputs and the output. The output layer includes prediction results of energy consumption in buildings.

In a classical ANNs model, a neuron is presented by the activating function as

(5) $$\begin{array}{c} f\left(xW+b\right). \end{array}$$

For the AANNs model, a neuron is presented by the activating function, where the affine transform is modified by using the efficient operator in

(6) $$\begin{array}{c} f\left(a•\left(x⋄W\right)+b\right), \end{array}$$

where • denotes the elementwise multiplication operator.

The neural network (NN) in each neuron represented by the activating function (equation (6) is called an additive NN. With regards to the training process of the AANNs model, the calculation of the argument derivative *f*(*a*•(*x*⋄*W*)+*b*) of the activating function is with parameters *W*, *a*, *b*, and inputs *x*.

(7) $$\begin{array}{c} \frac{\partial \left(a•\left(x⋄W\right)+b\right)}{\partial a}=\text{Diag}\left(x⋄W\right), \end{array}$$

(8) $$\begin{array}{c} \frac{\partial \left(a•\left(x⋄W\right)+b\right)}{\partial b}=I_{M}, \end{array}$$

(9) $$\begin{array}{c} \frac{\partial \left(a•\left(x⋄W\right)+b\right)}{\partial x_{i}}=\left[\begin{array}{c} a_{1}\left(\text{sign}\left(W_{i,1}\right)+2W_{i,1}\delta \left(x_{i}\right)\right)\\ a_{M}\left(\text{sign}\left(W_{i,M}\right)+2W_{i,M}\delta \left(x_{i}\right)\right) \end{array}\right]\approx a•\text{sign}\left(w_{i}\right), \end{array}$$

(10) $$\begin{array}{c} \frac{\partial \left(a•\left(x⋄W\right)+b\right)}{\partial W_{i,j}}=\left(a_{j}\left(\text{sign}\left(x_{i}\right)+2x_{i}\delta \left(W_{i,j}\right)\right)\right)e_{j}\approx a_{j}x_{i}e_{j}, \end{array}$$

where *a*, *b* ∈ R^*M*^ and *W* ∈ *R*^*dxM*^are the parameters of the hidden layer, *x* ∈ R^*d*^is the input of the hidden layer, *e*_*i*_ ∈ *R*^*M*^represents the *i*^th^ component of the standard basis of R^*M*^, *w*_*i*_represents the *i*^th^ column of *W*, sign(*w*_*i*_)=∑_*j*=1_^*M*^sign(*W*_*i*,*j*_)*e*_*j*_, for *i* = *1*,..., *M*, and *δ* represents the function of the Dirac delta.

The mentioned derivative was quickly computed using the following equation that was proposed in [32].

(11) $$\begin{array}{c} \frac{d}{dx}\text{sign}\left(x\right)=2\delta \left(x\right). \end{array}$$

In this study, the performance of AANNs was compared with baseline models that include the SVR models and ANNs model in predicting energy use in residential buildings. For the SVR model, the radial basis function (RBF) kernel and the polynomial (PL) kernel were used as kernel functions. The settings of these models were presented in the following section.

### 3.3. Model Settings and Implementation of the Proposed Model

Table 1 summarizes parameter information of the AI models used in this study. The investigated AI models include the SVR model with the PL kernel (SVR-PL), the SVR model with RBF (SVR-RBF), the ANNs model, and the AANNs model. These AI models were performed in the Weka platform [33] that is an open-source machine learning platform. The parameter settings of the AI models were implemented in the Weka [33].

**Figure 4:**
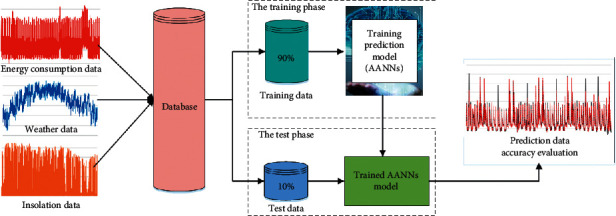
Implementation of the AANNs model.

Figure 4 shows the implementation of the AANN model. To develop the AI models and evaluate their performance, the original dataset has been divided into two subsets which are the training dataset (i.e., the first 90% of the dataset) and the test dataset (i.e., the last 10% of the dataset). The evaluation process consists of two steps that are the training phase and the test phase. The AI models were built using the training data which is accounting for 90% of the original data in the first phase. The test data, which is considered as the unseen data, are then fed into the trained AI model in the second phase to evaluate the effectiveness of the investigated AI models (i.e., the SVR, ANNs, and AANNs models).

During the evaluation process, the accuracy of the models was measured using common statistical indices that are mean absolute error (MAE), mean absolute percentage error (MAPE), RMSE, and correlation coefficient (*R*). These statistical indices were selected because they have been used to evaluate machine learning models in various studies [34]. The MAE is a measure of the difference between two continuous variables. In this study, the MAE is an average of the absolute errors between the hourly actual energy consumption values and the hourly predicted energy consumption values obtained by the AI models. Its formulation is presented as

(12) $$\begin{array}{c} \text{MAE}=\frac{1}{n}\overset{n}{\underset{i=1}{\sum }}\left|y-y^{\prime }\right|, \end{array}$$

where *y* represents the hourly actual net energy consumption data, *y'* is the hourly predicted net energy consumption data obtained by the AI models, and *n* is the number of data points in the sample.

The MAPE presents accuracy as a percentage. This index is commonly used for prediction problems, and in the proposed model, evaluation is due to its intuitive interpretation in terms of relative error. Equation (13) defines the MAPE calculation. The RMSE is a frequently used index of the differences between values forecasted by a prediction model and values measured. Its calculation is presented in equation (14).

(13) $$\begin{array}{c} \text{MAPE}=\frac{1}{n}\overset{n}{\underset{i=1}{\sum }}\left|\frac{y-y^{\prime }}{y}\right|, \end{array}$$

(14) $$\begin{array}{c} \text{RMSE}=\sqrt{\frac{1}{n}\overset{n}{\underset{i=1}{\sum }}{\left(y-y^{\prime }\right)}^{2}.} \end{array}$$

## 4. Experiment and Results

### 4.1. Data Source

**Figure 5:**
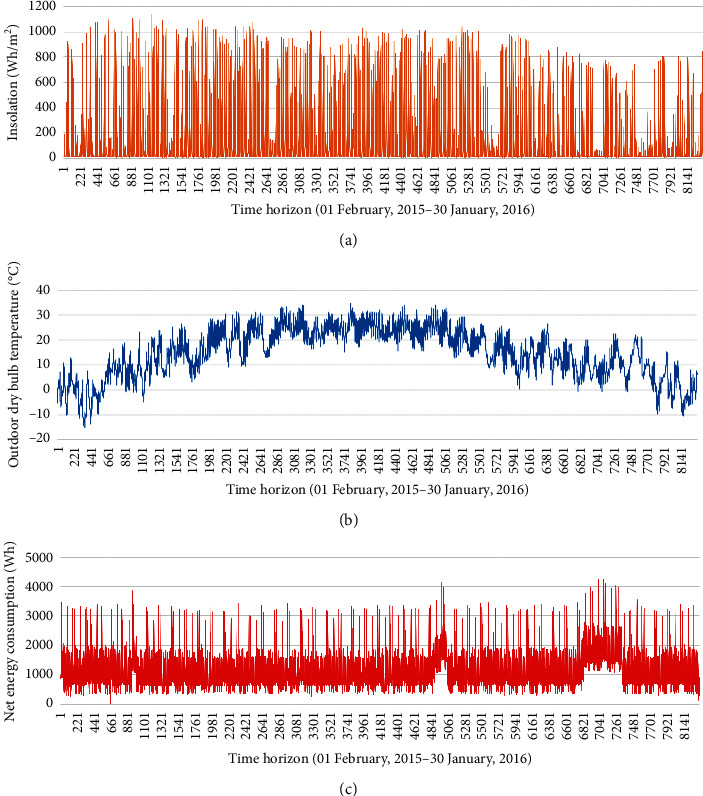
The hourly data profile for a year. (a) Hourly insolation profile. (b) Hourly profile of outdoor dry bulb temperature for a year. (c) Net energy consumption profile.

**Figure 6:**
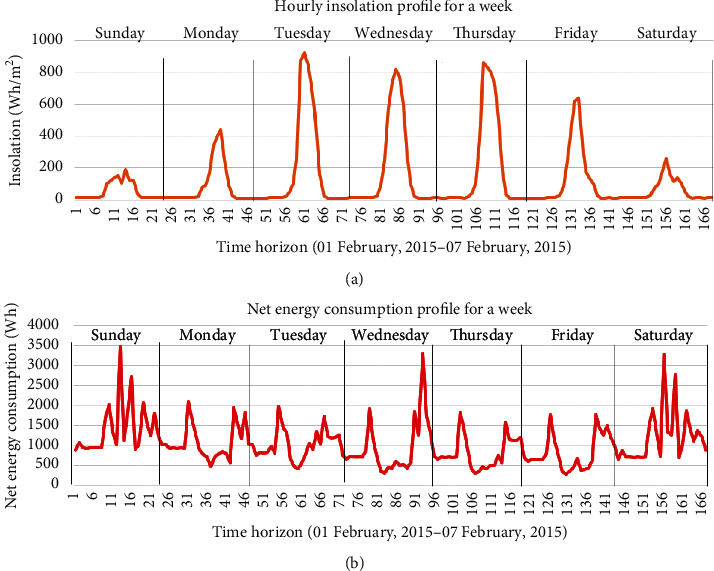
Hourly data profile of (a) insolation and (b) net energy consumption for a week.

Data used in this study were derived from the Net-Zero Energy Residential Test Facility at the National Institute of Standards and Technology (NIST) Engineering Lab [35, 36]. A solar photovoltaic system was used in this building as a renewable energy source. This data is open and free. The one-year dataset in hourly resolution was used to evaluate the proposed prediction model in this study.  Table 2 summarizes the input and output information that was used for training and testing AI models. Attributes of the dataset include the net building energy consumption, outdoor dry bulb temperature, insolation, day of the week, hour of the day. All data were collected hourly.  Figure 5 visualizes the outdoor dry bulb temperature, net energy consumption in the experimental building, and insolation profiles in the hourly resolution for a year. For providing readers with a clear look, Figure 6 presents the hourly insolation profile and hourly energy consumption profile in the building for a week.

### 4.2. Results and Discussion

The investigated AI models in this study include the SVR-PL, SVR-RBF, linear regression (LR), M5Rules, ANNs, and AANNs models. Their performance was assessed using a dataset that was recorded from a residential building with renewable energy. After the evaluation process, the predictive accuracy of these AI models is given in Table 3 via MAPE, MAE, RMSE, and *R* values regarding the training step and test step.

**Figure 7:**
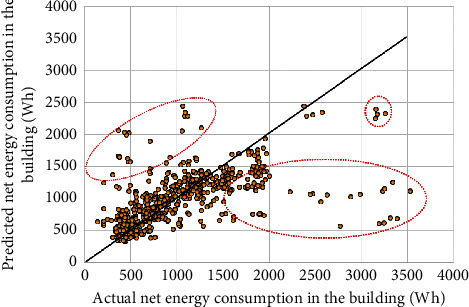
Prediction results by the SVR-PL model.

The SVR-PL model used the simple polynomial kernel as a kernel function for the prediction. Therefore, its performance did not look good in terms of accuracy indices, which are 26.18% and 28.60% in terms of the MAPE in the training phase and test phase, respectively. Similarly, the MAE and RMSE values obtained by the SVR-PL model were relatively high, up to 236.83 Wh and 430.69 Wh, respectively, for predicting residential building energy use profiles. The results of these statistical indices indicated that the SVR-PL was not effective in energy use prediction in residential buildings with renewable energy.  Figure 7 provides a visualization of the forecasted and recorded values of the net energy consumption obtained by the SVR-PL model. The diagonal line in Figure 7 indicates an absolute agreement between the forecasted and recorded values. The scatter plot in Figure 7 reveals that although most scatter points locate around the black, diagonal line, many points, that were in the three dashed red circles, were far from the absolute agreement. This means the SVR-PL model is still limited to capture an energy use profile.

**Figure 8:**
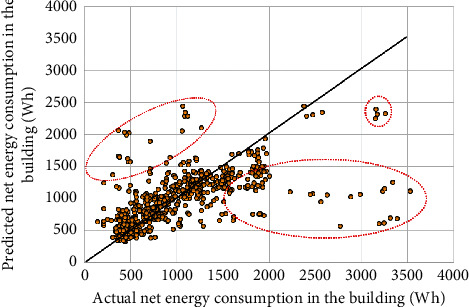
Scatter plot of prediction results by the SVR-RBF model.

When the RBF kernel was used as the kernel function in the SVR model, the performance of the SVR model was slightly enhanced in the prediction as compared to the SVR-PL model. The SVR-RBF model can yield predictive accuracy at 26.38% in the MAPE and 225.06 Wh in the MAE.  Figure 8 shows the scatter plot that compares the recorded net energy consumption in the experimental building and the predicted net energy consumption predicted by the SVR-RBF model. The scatter plot in Figure 8 presents that the SVR-RBF model underestimated energy consumption in the residential building which is illustrated at scattering points in the dashed red circle located above the black straight line. The scattering points in the dashed red circles located below the black straight line depicted over-estimated energy consumption by the SVR-RBF. The *R* values obtained by the SVR-RBF and SVR-PL models were lower than 0.7 in the training and test steps. Generally, two variants of the SVR models did not perform well in predicting the profiles of energy consumed in the experimental building.

**Figure 9:**
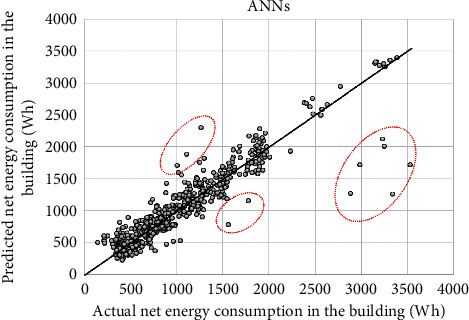
Prediction results by the ANNs model.

Figure 9 shows a comparing result between the observed net energy consumption data and energy consumption predicted by the ANNs model in the test phase. In terms of the MAPE, the ANNs models yielded 12.86% in the training phase and 14.68% in the test phase. The findings in Table 3 also revealed that the statistical indices of the ANNs model were 114.91 Wh in the MAE in the test phase. The *R* value achieved by the ANNs model was 0.932 in the test phase which depicts the good agreement between the actual and predicted data. These predictive results by the ANN model were better than those predicted by the SVR-PL and SVR-RBF models. As given in Table 3, the M5Rules model achieved good accuracy. Its accuracy indices were 14.2%, 112.44 Wh, 213.43 Wh, and 0.921 in the MAPE, MAE, RSME, and *R*, respectively. The LR model was ineffective in predicting energy patterns in buildings with low predictive statistical indices. The inherent linear characteristic of the LR limits its capability in modeling the nonlinear relationship between inputs and the predicted energy consumption.

**Figure 10:**
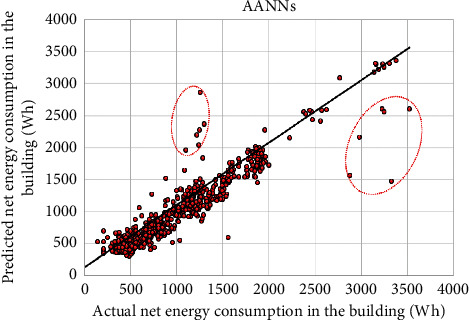
Scatter plots of actual and predicted net energy consumption in the building in the test step by the AANNs model.

**Figure 11:**
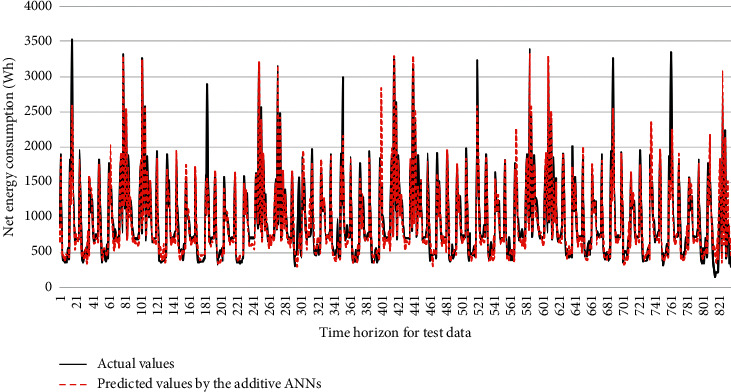
Actual and predicted net energy consumption by AANNs in test phase.

Table 4 provides the performance comparison among the investigated ML models. The proposed AANNs model achieved a good predictive accuracy, in which its statistical measures were 14.04% in the MAPE, 111.98 Wh in the MAE, 188.68 in the RMSE, and 0.940 in the *R* for predicting hourly net energy consumption during the test phase.  Figure 10 shows the prediction values of energy consumption in the residential building. Most scatter points were close to the black line which means the AANNs model was effective in forecasting the hourly net energy consumption in the building with the renewable energy source. Besides, Figure 11 plots actual and predicted values of net energy consumption by AANNs in the test phase over the time horizon.

**Figure 12:**
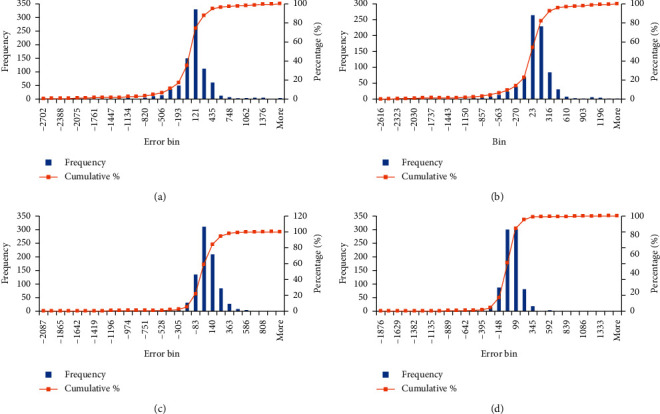
Prediction error histogram by the prediction models. (a) Error histogram by the SVR-PL model. (b) Error histogram by the SVR-RBF model. (c) Error histogram by the ANNs model. (d) Error histogram by the AANNs model.

The performance comparison among the ML models in Table 4 depicts that the AANNs model was the most effective forecasting model in terms of all performance indices of the MAPE, MAE, RMSE, and *R*. The proposed AANNs model obtained the lowest MAPE with 14.04%, followed by the ANNs model with the MAPE of 14.68% and SVR-RBF with the MAPE of 26.38%.  Figure 12 presents the prediction error histogram produced by the SVR-PL model (Figure 12(a)), the SVR-RBF model (Figure 12(b)), the ANNs model (Figure 12(c)), and the proposed AANNs model (Figure 12(d)). Compared to the predictive performance of the SVR-PL model, the AANNs model can significantly improve the predictive accuracy which was about 103.75% in the MAPE, 111.50% in the MAE, and 128.26% in the RMSE. Similarly, the AANNs outperformed significantly the SVR-RBF models in the residential building energy consumption prediction. Because the AANNs model is an enhanced version of the ANNs, its performance was better than those of the ANN model. Compared to the ANNs model, accuracy improvement percentages by the AANNs model were 4.6% in the MAPE, 2.61% in the MAE, and 5.81% in the RMSE. The comparisons in Table 4 confirmed the outperformance of the AANNs models to other models.

The Wilcoxon signed-rank test is a nonparametric test in which two paired samples were compared to evaluate a significant difference between two population means. This statistical test has been used in [28, 37, 38] to confirm the significance of the accuracy enhancement. Thus, the Wilcoxon signed-rank test was used in this study. The Wilcoxon signed-rank test was used for pair comparison between prediction results by the AANNs model with those obtained by the SVR-PL, SVR-RBF, and ANNs models, respectively. The statistical test results depicted that the computed *p* value was lower than the significance level alpha of 0.05. Thus, the null hypothesis H_0_ was rejected, and the alternative hypothesis *H*_a_ was accepted. Therefore, the significant difference was confirmed between the performance of the AANNs models and other compared models.

## 5. Conclusions

Energy efficiency is one of the most concerning topics within academic researchers and decision-makers in the energy sector. An energy consumption prediction in a building is the basis for optimizing building performance and reducing energy costs. This study proposed additive artificial neural networks (AANNs) that can accurately predict energy consumption in buildings concerning historical data of energy use and weather conditions. This study also compared the performance of the AANNs with other ML models such as the support vector regression with the polynomial kernel function (SVR-PL), the support vector regression with the radial basis function kernel function (SVR-RBF), and the artificial neural networks (ANNs). Their performance was assessed using a one-year dataset in the hourly resolution that was recorded from a residential building with renewable energy sources.

The proposed AANNs model achieved a good predictive accuracy in which its statistical measures were 14.04% in the mean absolute percentage error (MAPE) and 111.98 Watt-hour in the mean absolute error (MAE) for predicting hourly net energy consumption. The AANNs model was the most effective forecasting model among the investigated AI models. Compared to the support vector regression (SVR), the AANNs model can significantly improve the predictive accuracy by about 103.75% in the MAPE and 111.50% in the MAE. Similarly, compared to the ANNs model, accuracy improvement percentages by the AANNs model were 4.6% in the MAPE and 2.61% in the MAE. Thus, the AANNs model was recommended as an effective AI-based model for predicting net energy consumption in residential buildings with a solar photovoltaic system.

The contributions of this study include (1) collection of building energy use profiles in hourly resolution and their associated weather data, (2) investigation of the potential power of artificial intelligence techniques in forecasting future energy consumption in buildings, and (3) development of the effectiveness and capability of the AANNs in the prediction of building energy consumption.

## Figures and Tables

**Table 1:** Settings of investigated AI models.

Model	Settings
SVR-PL	Classifier = SMOreg; *c* = 1.0; filterType = normalize training data; kernel = PolyKernel; exponent = 1.0
SVR-RBF	Classifier = SMOreg; *c* = 1.0; filterType = normalize training data; kernel = RBFKernel; gamma = 0.01
LR	AttributeSelectionMethod = *M*5 method; eliminateColinearAttributes = true, ridge = 10^−8^
M5Rules	BuildRegresionTree = false, unpruned = false; useUnsmoothed = false
ANNs	HiddenLayers = *a*; learningRate = 0.3; momentum = 0.2; normalizeAttributes = true; trainingTime = 500
AANNs	Classifier = AdditiveRegression_MultilayerPerceptron; numIterations = 10; shrinkage = 1.0; hiddenLayers = *a*; learningRate = 0.3; momentum = 0.2; normalizeAttributes = true; trainingTime = 500

**Table 2:** Data attributes for model evaluation.

Symbol	Parameter	Unit	Value
Input			
*X*1	Day of the week	—	Monday, Tuesday, Wednesday, Thursday, Friday, Saturday, Sunday
*X*2	Hour of the day	—	0, 1, 2,…, 21, 22, 23
*X*3	Isolation	Wh/m^2^	
*X*4	Outdoor dry bulb temperature	°C	
Y_historical	Historical building energy consumption	Wh	
Output			
*Y*	Future building energy consumption	Wh	

**Table 3:** Predictive accuracy of the machine learning models for building energy consumption.

ML models	Accuracy measures in the training step. Training data (*N* = 7493, 90%)				Accuracy measures in the test step. Test data (*N* = 835, 10%)			
	MAPE (%)	MAE (Wh)	RMSE (Wh)	*R*	MAPE (%)	MAE (Wh)	RMSE (Wh)	*R*
SVR-PL	26.18	240.69	438.45	0.653	28.60	236.83	430.69	0.622
SVR-RBF	24.13	226.58	412.98	0.698	26.38	225.06	412.53	0.659
LR	30.74	265.67	409.80	0.586	36.74	275.68	416.64	0.654
M5Rules	11.96	104.26	172.77	0.953	14.20	112.44	213.43	0.921
ANNs	12.86	106.80	170.28	0.955	14.68	114.91	199.64	0.932
AANNs	12.78	106.88	165.15	0.961	14.04	111.98	188.68	0.940

**Table 4:** Performance comparison among machine learning models.

ML models	Accuracy measures in the test step				Improvement percentage (%)			
	MAPE (%)	MAE (Wh)	RMSE (Wh)	*R*	MAPE	MAE	RMSE	*R*
SVR-PL	28.60	236.83	430.69	0.622	103.75	111.50	128.26	33.83
SVR-RBF	26.38	225.06	412.53	0.659	87.96	100.98	118.64	29.89
LR	36.74	275.68	416.64	0.654	161.78	146.19	120.82	30.48
M5Rules	14.20	112.44	213.43	0.921	1.14	0.41	13.12	2.00
ANNs	14.68	114.91	199.64	0.932	4.60	2.61	5.81	0.85
AANNs	14.04	111.98	188.68	0.940				

## Data Availability

The data used to support the findings of this study are included within the article.
